# The effect of dexamethasone and bupivacaine on postoperative pain and nausea and vomiting by preperitoneal nerve blocking in laparoscopic cholecystectomy: a randomized clinical trial

**DOI:** 10.1097/MS9.0000000000002338

**Published:** 2024-07-31

**Authors:** Mohammad Eslamian, Erfan Sheikhbahaei, Ali Esparham, Hamidreza Zefreh, Amirhossein Fasahat, Alireza Firouzfar, Hamid Talebzadeh

**Affiliations:** a Department of Surgery, School of Medicine; b Isfahan Minimally Invasive Surgery and Obesity Research Center, Alzahra University Hospital, School of Medicine; c School of Medicine, Isfahan University of Medical sciences, Isfahan; d Student Research Committee, Faculty of Medicine, Mashhad University of Medical since, Mashhad, Iran

**Keywords:** bupivacaine, corticosteroids, dexamethasone, laparoscopic cholecystectomy, postoperative nausea, postoperative pain

## Abstract

**Background:**

This study aims to examine the effects of preperitoneal administration of dexamethasone and bupivacaine surrounding laparoscopic trocars on postoperative pain (POP) and nausea and vomiting (PONV) in patients undergoing laparoscopic cholecystectomy (LC).

**Method:**

In this randomized triple-blinded trial with a 1:1 randomization ratio, 104 patients with chronic cholecystitis were candidates for elective LC. A total of 40 mg (8 ml) of bupivacaine was mixed with 8 mg (2 ml) of dexamethasone or normal saline. The solution was injected preperitoneally via an 18G needle parallel and lateral to trocars until a bulge in the interior surface of the parietal peritoneum was observed by the camera. Primary outcomes were the severity of POP based on 0–10 Likert visual analog scale (VAS) and rates of PONV and secondary outcomes were rate of postoperative opioid usage and any side-effects.

**Result:**

The mean VAS score was significantly lower in the dexamethasone group (3.5 vs. 6.2, *P*<0.001). The dexamethasone group had 46.2% and 26.9% lower rates of nausea and vomiting after LC compared to the other group (*P*=0.001 and 0.015, respectively). Postoperative opioid use was lower in the dexamethasone group, but its difference was insignificant (*P*=0.3).

**Conclusions:**

Preperitoneal dexamethasone injection around laparoscopic trocars may lower the intensity of POP and PONV rates. Perioperative local corticosteroids can be used as an effective, available, and inexpensive analgesic and antiemetic prevention for laparoscopic procedures.

## Introduction

Highlights

- Preperitoneal dexamethasone injection around laparoscopic trocars may lower the intensity of postoperative nausea.
- Preperitoneal dexamethasone injection around laparoscopic trocars may lower the intensity of postoperative pain.

Laparoscopic cholecystectomy (LC) is the preferred surgical procedure for treating cholelithiasis, cholecystitis, and some types of cholecystic polyps^1,2^. The advantages of LC compared with open cholecystectomy are less postoperative pain (POP), less metabolic interference and morbidity, and faster recovery^2–4^. Nevertheless, LC is associated with postoperative surgical site pain and postoperative nausea and vomiting (PONV), both of which can have a detrimental impact on patient satisfaction and perhaps lead to severe outcomes^5,6^.

POP impacts operation outcomes, patient satisfaction, development of hyperventilation, tachycardia, alveolar hypoventilation, transition to chronic pain, insomnia, poor wound healing, and increased mortality and morbidity^7^. In addition, it was shown that PONV occurs in around 30% of all types of surgical procedures and in 52–80% of patients after LC^8^. Various risk factors have been identified for PONV, including a prior history of nausea and vomiting, being female, experiencing motion sickness in the past, using opioids, and undergoing prolonged surgical procedures^8,9^. Therefore, finding proper prevention for POP and PONV can mitigate complications and improve quality of life.

Glucocorticoids such as dexamethasone are popular for their analgesic, antiemetic, and anti-inflammatory effects by impacting endogenous prostaglandin and opioid production mechanisms^10–12^. Currently, corticosteroids are shown to have some impacts on nerve endings and therefore some of their analgesics may be explained by blocking sensory nerve signaling^13^. In addition, it was noted that the prophylactic antiemetic effect of dexamethasone can be enhanced by other antiemetics in comparison to dexamethasone alone^14^. However, the combined effect of local injection of dexamethasone with bupivacaine, which is usually used as a common local analgesic, was not fully investigated in patients undergoing LC. Most studies used systemic or peritoneal administration of corticosteroids, and a few of them injected dexamethasone at the exact site of intervention, which is between the skin and parietal peritoneum. Therefore, this study aims to investigate the outcomes of perioperative local injection of dexamethasone plus bupivacaine vs. bupivacaine alone on POP and PONV in LC.

## Material and methods

### Trial design

This parallel triple-blinded randomized controlled trial was carried out in our university-affiliated hospital between November 2021 and April 2022. Our institutional review board approved the ethical considerations and the study commenced after receiving its registration number from our national database for the registry of clinical trials. This study commenced after receiving its approval from the department of medical ethics of Isfahan University of Medical Sciences with the registration number “IR.MUI.MED.REC.1400.546”. The protocol of this study was approved by Iran registry of clinical trials (“IRCT20211008052699N1”). Prior to surgery, all patients provided informed consent.

### Participants

The inclusion criteria were all elective LC with a clinical diagnosis of chronic cholecystitis, which cholelithiasis confirmed with ultra-sonography and have American Society of Anesthesiology (ASA) physical status I and II, without allergic reaction to local anesthetics or corticosteroids. Patients who denied informed consent and with an age lower than 18 years old, history of addiction, gallbladder fistula to the duodenum, gangrene or Mirizzi’s syndrome diagnosed during the surgery, converting LC to open surgery, type 2 diabetes mellitus, presence of acute cholecystitis, pregnancy, hepatobiliary malignancies, compromised systemic illness, portal hypertension, and any wounds on the abdomen were not included in this study.

### Intervention

**Figure 1:**
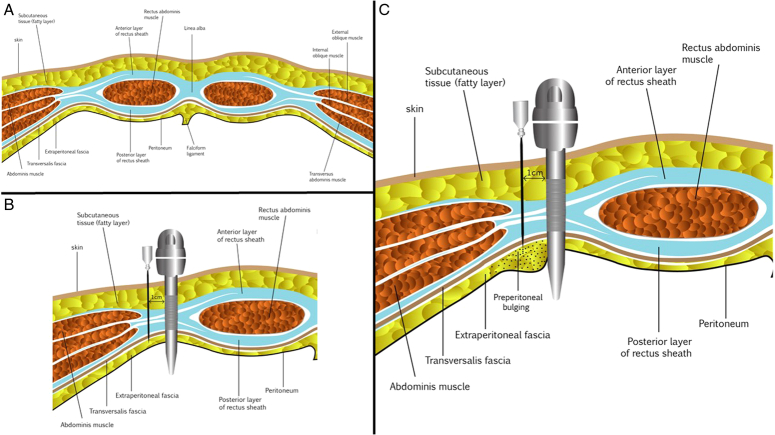
The method of preperitoneal nerve blocking with dexamethasone and bupivacaine injection.

After conducting electrocardiographic monitoring, blood pressure measurement, pulse oximetry, capnography, and administering 10 ml/kg of crystalloids to all participants for hydration, general anesthesia was started. The induction process included the use of midazolam (0.05 mg/kg), fentanyl (2 µg/kg), sodium thiopental (5 mg/kg), and atracurium (0.5 mg/kg). The same surgical team performed all LC using 4 laparoscopic ports (10 mm for periumbilical and epigastric, 5 mm for the midclavicular and anterior axillary ports). A total of 40 mg (8 ml) of bupivacaine was mixed with 8 mg (2 ml) of dexamethasone, which was according to the previous studies^9,15,16^. Afterward, the solution was diluted with 5 ml of distilled water, and then 5 and 2.5 ml of the solution was injected at each 10 mm and 5 mm port, respectively. For the control group, 2 ml normal saline was mixed with bupivacaine. An 18-gauge needle was inserted parallel to the trocar’s pathway and perpendicular to the skin and 5 mm lateral to the trocars (Fig. 1). The solution was injected preperitoneally until a bulge in the interior surface of the parietal peritoneum was observed by the camera (as shown in the supplementary video 1, Supplemental Digital Content 2, http://links.lww.com/MS9/A575).

### Sample size

Considering alpha=0.05, beta=0.2, and what has been reported for the mean and standard deviations of VAS 24 hours after LC and respecting 20% predicted missed cases a total of 104 patients was calculated to be sufficient for this study^17^.

### Outcome

An expert-blinded medical profession entered the information and evaluated the study outcomes. Age, gender, medical history, weight, height, BMI, and history of pancreatitis, biliary colic, and acute cholecystitis were extracted from the patient’s medical records. The primary outcomes were the POP intensity and frequency of PONV, and the secondary outcome was postoperative opioid use (pethidine) on the next day after surgery. The POP intensity was measured by the visual analog scale (VAS) with a 0–10 Likert scale and was evaluated 24 h after finishing the surgery^18^. In addition, nausea was described as a subjective sensation characterized by an urge to vomit, unaccompanied by any forceful muscular contractions for expulsion. Vomiting was defined as the act of forcefully expelling the contents of the stomach involuntarily. Additionally, the presence of PONV was acknowledged if the patient reported either nausea or vomiting occurrence during the first 24 h after LC. Our pain management protocol was based on VAS and systematic infusion of acetaminophen. Any patient in pain received one gram of acetaminophen every 6 h. In case of resistance or severity of more than mild pain on VAS, a single shot of intramuscular pethidine (50 mg) was administered.

### Randomization and blinding

An analyst who was blinded to the patient’s confidential information, and pre and postoperative outcomes prepared and conducted the randomization process by computer-generated random number. ﻿A consecutive randomization number belonged to each enrolled patient. The list was concealed within sequentially numbered, sealed envelopes, which were opened in order following the acquisition of patient consent. A blinded pharmacist of our center purchased the medication and re-labeled the vials to dexamethasone (A) and normal saline (B).

For blinding the surgeon from each patient’s group, on the surgery due date, one of the trained operating room technicians based on randomized numbers, prepared the medicine for the group in which the patient was categorized and then the surgeon injected it blindly under laparoscopic camera before ending the operation. An equal volume of dexamethasone or normal saline combined with bupivacaine was pulled into a syringe, and the combination was identified only based on groups A (containing dexamethasone plus bupivacaine) and B (normal saline with bupivacaine).

### Statistical analysis

The categorical and numerical data are presented as frequency (percentage) and mean ± standard deviation, respectively. The χ^2^ test was employed to assess the categorical data. Additionally, the student *t*-test was used for normally distributed numerical data, whereas the Mann–Whitney U test was used for non-normally distributed numerical data. All analyses were done by IBM SPSS version 26. A *P* value below 0.05 was deemed to be statistically significant.

## Results

**Figure 2:**
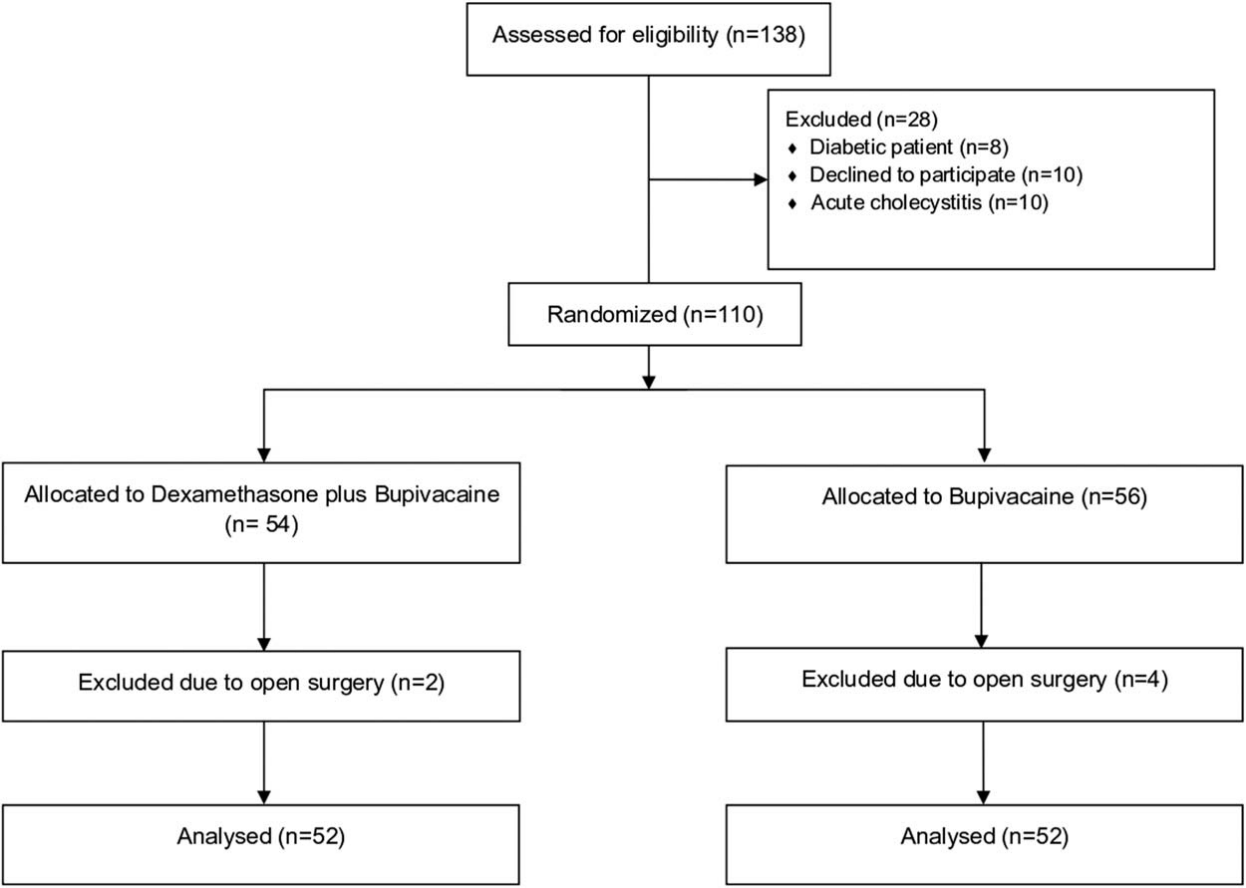
The CONSORT flowchart diagram of included patients.

Finally, a total of 104 patients were enrolled in this study each with 52 patients. The CONSORT, Supplemental Digital Content 1, http://links.lww.com/MS9/A574, flowchart diagram is presented in Fig. 2. The baseline characteristics of patients are shown in Table 1.

**Table 1:** Demographic and preoperative patients’ characteristics of each group

Variables	Group A (Dexamethasone+Bupivacaine) *n*=52	Group B (Bupivacaine alone) *n*=52
Age (years)	46.31±10.6	42.94±11.85
Male sex, *n* (%)	18 (34.6)	14 (26.9)
BMI (kg/m^2^)	27.38±4.41	28.89±5.22
History of pancreatitis, *n* (%)	14 (25.9)	10 (19.2)

Nominal variables are presented as number of patient (percentage) and numerical variables are presented as mean ± standard deviation.

All the *p* values are not significant with the level > 0.05.

**Figure 3:**
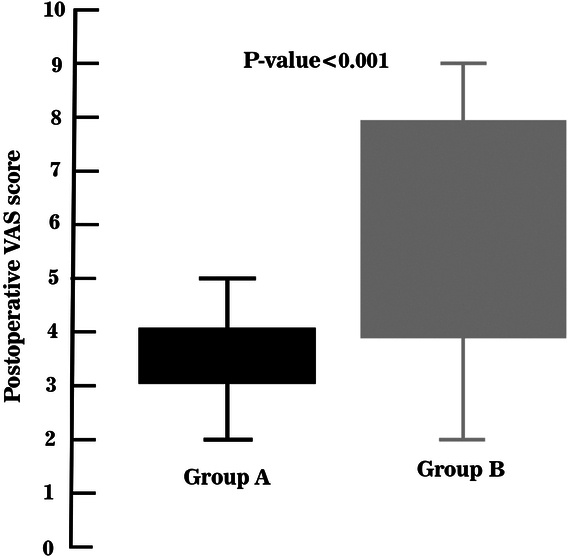
The comparison of postoperative pain intensity between dexamethasone+bupivacaine and bupivacaine alone groups. The VAS for group A and B is 3.38±1.91 and 6.23±2.02 (Mean±SD) respectively. VAS, visual analog scale.

Based on reported VAS, bupivacaine alone group had a significantly higher intensity of pain 24 h after LC (6.23**±**2 vs. 3.38**±**1.9, *P*<0.001). Figure 3 demonstrates POP intensity in both groups. Mild pain category was significantly higher in dexamethasone group (*P*<0.001); however, severe pain was more experienced in the other (*P*=0.001). Table 2 shows the categorized postoperative intensity based on the VAS score.

**Table 2:** Postoperative pain intensity category based on visual analog scale

Pains severity	Group A (Dexamethasone+Bupivacaine) *n*=52	Group B (Bupivacaine alone) *n*=52	*P*
No pain (0), *n* (%)	0	0	—
Mild (1,2,3), *n* (%)	34 (63)	6 (11.5)	<0.001
Moderate (4,5,6,7), *n* (%)	18 (37)	28 (53.8)	0.048
Severe (8,9,10), *n* (%)	0	18 (34.6)	<0.001

Nominal variables are presented as number of patient (percentage).

All the *p* values are significant with the level < 0.05.

**Figure 4:**
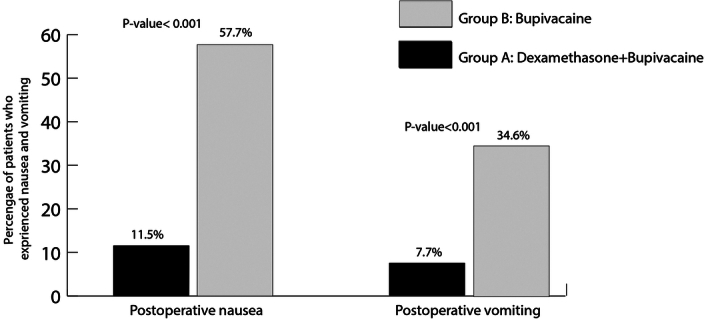
The comparison of postoperative nausea and vomiting between dexamethasone+bupivacaine and bupivacaine alone groups.

The number of patients who experienced postoperative nausea and vomiting were significantly lower in dexamethasone group (with absolute risk reduction=46.2%, *P*=0.001, number needed to treat=2.1; absolute risk reduction=26.9%, *P*=0.015, number needed to treat=3.7, respectively). Figure 4 presents the PONV episodes during the first 24 hours after LC in both groups. Although the rate of postoperative opioid use was lower in dexamethasone group, its difference was not significant (11.5% vs. 22.2%, respectively, *P*=0.3). Finally, no side-effects from either group was reported.

## Discussion

The results of the current trial showed that preperitoneal injection of dexamethasone+bupivacaine around laparoscopic ports can reduce the POP intensity and PONV rate after LC. POP usually results from visceral pain, incision wounds, and diaphragm irritation, which can lead to shoulder pain and pneumoperitoneum^19,20^. In 26–41% of LC patients, POP is the major reason for prolonged hospital stay and convalescence period^17,21^. It has been demonstrated that small doses of corticosteroids can alleviate POP, decrease fatigue, improve patients’ mood, and increase appetite^22^. Locally infiltrated dexamethasone can decrease cytokines production and proinflammatory factors in impaired tissue by lipocortin 1, which is a glucocorticoid inducible protein that has a potential endogenous mediator role in the anti-inflammatory activities of glucocorticoids. In addition, inhibiting phospholipase enzyme by plasma dexamethasone absorbed from local tissue and decreasing the level of sensory nerve proteins that are involved in pain sensation are other potential mechanisms^23–25^. Furthermore, it has been shown that local dexamethasone decreases sensory nerve conduction by blocking neurotransmitter release from terminal axons^13^. While most of the previous studies used intravenous or intraperitoneal injections of dexamethasone, the current trial used local injections of dexamethasone at the laparoscopic ports. Since different methods of dexamethasone administration have different distribution, absorption, and half-life, local injection of dexamethasone at the surgical site may provide a repository effect with slow absorption and prolonged duration in comparison to systemic administration^24^. Furthermore, the local dexamethasone may show a second phase of action by being absorbed to systemic circulation and bringing a more extended effect similar to intravenous administration^26^; however, this hypothesis couldn’t be investigated in our trial. The results of our study are in line with the previous with a bold difference in the mode of administering dexamethasone. Intravenous dexamethasone reduced postoperative VAS, length of hospital stays, and the need for postoperative analgesics in LC patients^21^. Moreover, intraperitoneal administration of dexamethasone demonstrated a decrease in the POP during the first hours after LC and lowered the postoperative analgesic requirement^27^. Nevertheless, Zahra *et al*. reported intraperitoneal bupivacaine+magnesium sulfate reduced POP and postoperative analgesics consumption more in comparison to bupivacaine+dexamethasone in LC patients^28^. However, unlike our study, they did not exclude patients with addiction, which can be an important confounding factor and the dose of dexamethasone was 8 mg, which was lower than our study. In Mohtadi *et al*.’s^29^ study, although the VAS score was significantly lower after 2, 6, and 12 h in patients receiving dexamethasone, there was no significant difference in VAS score between the two groups after 24 h. However, these discrepancies can be due to lower doses of dexamethasone (0.1 mg/kg) and different routes of injection (intravenous).

Furthermore, PONV lead to increased risk of postoperative wound dehiscence, gastric aspiration, electrolyte imbalance, and hemorrhage in patients who underwent LC^30^. Furthermore, it can extend the post-anesthesia care unit and overall health care costs^14^. Therefore, there is a need for prophylaxis, especially in high-risk patients such as women, with a history of motion sickness, and patients on postoperative opioids^31^. The following risk factors have been proposed: intraoperative use of nitrous oxide, carbon dioxide insufflation, peritoneum scratching, and increasing blood pressure in the peritoneal cavity^21^. Since PONV has a multifactorial nature, full prevention of PONV after LC is difficult. Nevertheless, it was shown that a combination of antiemetic drugs can prevent PONV in severe cases^14^. The efficacy of dexamethasone as an antiemetic medication is shown in patients who underwent laparoscopic surgeries^31,32^. The antiemetic effect of dexamethasone can be explained by several hypotheses; prostaglandin antagonism and inhibition of 5-HT (serotonin) through decreasing tryptophan or preventing its receptor from being activated in the gastrointestinal tract by its anti-inflammatory nature^12^. Also, dexamethasone can significantly enhance the efficacy of other antiemetic drugs by increasing their receptor sensitivity^33^. Additionally, it can potentially enhance the release of endorphins or amplify the effectiveness of other antiemetic medications by heightening their receptor sensitivity^33^. The DREAMS study demonstrated that administering 8 mg of intravenous dexamethasone during the induction of anesthesia effectively decreased the occurrence of PONV within 24 h. Additionally, the need for rescue antiemetics was reduced for 72 h after surgery. Importantly, this reduction in PONV was observed in patients who received either laparoscopic or open bowel surgery, without any notable increase in side events^34^. It was shown that the combination of dexamethasone and other antiemetic medications can lead to a significantly more preventing effect on PONV and fewer rescue antiemetics in comparison to single antiemetic medications in patients who underwent LC^14^. Elhakim *et al*.^35^ suggested preoperative dexamethasone alone or in combination with other antiemetics reduced the incidence of PONV in patients undergoing LC. All of these studies are in line with our findings regarding the effectiveness of local dexamethasone for reduction in PONV rates in LC patients.

The findings of this trial should be interpreted cautiously due to our limitations; the VAS score was only measured after 24 h, postoperative analgesic consumption has not been measured precisely, and the side effect of dexamethasone was not investigated. However, earlier research showed no significant side-effects following a single dose of dexamethasone^21^. In addition, relatively small sample size and the absence of longer follow-up are the other limitations of our study.

Further studies on the effect of the combination of dexamethasone with other analgesics and antiemetics in a larger sample size and with longer follow-up are recommended. At last, a comprehensive systematic review and meta-analysis can clarify the antiemetic and analgesic properties of local dexamethasone in LC patients.

## Conclusion

Preperitoneal dexamethasone injection around laparoscopic trocars may lower the intensity of postoperative pain and nausea and vomiting rates after laparoscopic cholecystectomy. These results can potentially bring a reduction in hospital stay, financial costs, morbidity, and mortality rate, and finally, improve the quality of life of patients.

## Ethical considerations

This study commenced after receiving its approval from the department of medical ethics of Isfahan University of Medical Sciences with the registration number “IR.MUI.MED.REC.1400.546”. All patients received the highest standard medical care needed according to the latest guidelines in our hospital. Informed written consent obtained from all the participants before starting the trial. Iran registry of clinical trials approved the study protocol. (“IRCT20211008052699N1”).

## Consent

Written informed consent was obtained from the patient for publication and any accompanying images. A copy of the written consent is available for review by the Editor-in-Chief of this journal on request.

## Source of funding

None.

## Author contribution

M.E., A.F., H.T.: conceptualization. M.E., E.S., A.E., A.F., H.T.: methodology. E.S., H.Z.: software. M.E., A.F., H.T.: validation. E.S., A.E., H.Z., A.F.: formal analysis. M.E., A.F., H.T.: investigation. M.E., A.F., H.T.: resources. M.E., A.F., H.T.: data curation. Writing - Original Draft: all authors. Writing - Review & Editing: all authors. E.S., A.E., H.Z., A.F.: visualization. M.E., A.F., H.T.: supervision. M.E., E.S., A.E., H.Z., A.F., A.F., H.T.: project administration. M.E., A.F., H.T.: funding acquisition.

## Conflicts of interest disclosure

There is no conflict of interest to be declared by all the authors.

## Research registration unique identifying number (UIN)

IR.MUI.MED.REC.1400.546.

## Guarantor

Mohammad Eslamian.

## Data availability statement

Data are available upon reasonable request.

## Provenance and peer review

Not commissioned, externally peer-reviewed.

## Supplementary Material
